# Disparities in Pediatric Firearm Injury Care: A Comparison of Chronic Illness Pathways

**DOI:** 10.1007/s11121-025-01865-0

**Published:** 2026-01-02

**Authors:** Kelsey Gastineau, Velma Murry, Rasheedat Fetuga, Sabrina Carro, Regan Williams, Krista R. Mehari

**Affiliations:** 1 Department of Pediatrics, Monroe Carell Jr. Children’s Hospital at Vanderbilt, Vanderbilt University Medical Center, Nashville, TN USA; 2 Department of Health Policy, Vanderbilt University Medical Center, Nashville, TN USA; 3 Gideon’s Army, Nashville, TN USA; 4 Department of Surgery, Le Bonheur Children’s Hospital, The University of Tennessee Health Science Center, Memphis, TN USA; 5 Department of Psychology and Human Development, Vanderbilt University, Nashville, TN USA; 6 Department of Pediatrics, Medical College of Wisconsin, Milwaukee, WI USA

**Keywords:** Firearm injury prevention, Clinical practice guidelines, Health equity, Trauma-informed care, Pediatric trauma

## Abstract

Pediatric firearm injuries represent a leading cause of morbidity and mortality in the USA, yet clinical care models for youth with firearm injuries remain fragmented and unstandardized. Unlike other serious pediatric conditions such as asthma and cancer, which benefit from established interdisciplinary care pathways, firearm injuries are often treated as isolated events without structured follow-up or coordinated support systems. This absence of standardized clinical guidelines reflects broader systemic biases in how these injuries are perceived and managed within health care. Drawing on the Injury Equity Framework and established chronic disease management models, we propose comprehensive, trauma-informed clinical guidelines for pediatric firearm injury care spanning primary, secondary, and tertiary prevention. These guidelines emphasize universal firearm safety screening integrated into routine pediatric visits, standardized protocols for acute trauma care with automatic interdisciplinary team activation, and structured longitudinal follow-up addressing medical, behavioral health, and social needs. By comparing current fragmented approaches to evidence-based care pathways used in asthma and cancer management, we identify critical gaps in continuity and comprehensiveness of firearm injury care and offer specific, actionable recommendations to close these gaps.

**Supplementary Information:**

The online version contains supplementary material available at 10.1007/s11121-025-01865-0.

## Introduction

Firearm injuries have become the leading cause of death among children and adolescents in the USA, surpassing motor vehicle collisions in 2020 and remaining the top cause of pediatric mortality through 2024 (Centers for Disease Control & Prevention, 2024). Each year, approximately 3500 children (0–17) die from firearm injuries, and an additional 15,000 survive with often life-altering consequences (Goldstick et al., 2022). These injuries encompass both intentional violence—including interpersonal assaults and self-harm—and unintentional shootings, with substantial variation across developmental stages and geographic contexts.

The physical and psychological sequelae of pediatric firearm injuries extend far beyond the acute hospitalization. Survivors face elevated risks for post-traumatic stress disorder, chronic pain, substance use disorders, and long-term functional impairments that affect educational attainment, employment, and quality of life (Ranney et al., 2019; Vella et al., 2020). In the year after injury, pediatric firearm injury survivors demonstrate significantly elevated health care utilization compared to matched controls, with increased emergency department visits, hospitalizations, and outpatient encounters reflecting ongoing medical and behavioral health needs (Gastineau et al., 2024). Family members and peers exposed to firearm violence experience secondary trauma, creating ripple effects throughout communities (Song et al., 2023). The economic burden is also substantial, with direct medical costs exceeding $1 billion annually and indirect costs—including lost productivity and reduced quality of life—reaching into the tens of billions (Fowler et al., 2017).

Critically, these burdens are not distributed equitably. Black and Latino youth experience disproportionately high rates of fatal and nonfatal firearm injuries compared to their White peers, reflecting longstanding patterns of structural racism and inequitable resource distribution (Formica, 2021; Sakran et al., 2020). Youth in neighborhoods characterized by concentrated poverty, residential instability, and limited economic opportunity face substantially elevated risk, independent of individual-level characteristics (Jacoby et al., 2018; Pino et al., 2023). These disparities emerge not from inherent differences in communities or individuals, but from decades of policy decisions that have systematically created segregated neighborhoods and then disinvested in, and divested resources from, predominantly Black and Latino neighborhoods (Bailey et al., 2021; Formica, 2021). Redlining practices denied generations of families access to wealth-building through homeownership (Mehranbod et al., 2022; Owens et al., 2024). Mass incarceration destabilized family structures and removed economic contributors from communities. The removal of social services—including mental health care, youth programming, and violence prevention infrastructure—left communities without essential supports (Bailey et al., 2021). The militarization of policing further fostered mistrust between institutions and residents while failing to address root causes of violence (Jacoby et al., 2018; Pino et al., 2023).

Similar structural inequities that expose children to firearm violence also influence the medical care they receive after injury (Danielson, 2022). The health care system itself can reinforce these disparities through provider biases, institutional neglect, and the pervasive framing of firearm injuries as issues of criminality rather than public health (Sakran et al., 2020; Slopen et al., 2024). Unlike children diagnosed with asthma or cancer—whose illnesses are met with empathy, structured treatment, and community support—children who survive firearm injuries are often viewed as complicit in their own harm (Sim et al., 2021). This “blame the patient” framing influences provider attitudes and care decisions, shaping whether a child is seen as a victim in need of intervention or as someone undeserving of comprehensive care. Research demonstrates that Black and Latino youth receive lower-quality care across numerous pediatric conditions, including inadequate pain management, fewer specialty referrals, and limited access to rehabilitative services (Goyal et al., 2015; Slopen et al., 2024). When these biases intersect with the stigma attached to firearm injury, the quality gap widens further. Moreover, hospitals serving predominantly Black and Latino communities often have fewer resources, less robust specialty services, and more limited behavioral health support compared to those serving predominantly White communities and those from predominately affluent populations (Haider et al., 2013). The spillover consequences are profound: neglecting necessary follow-up services, failing to re-integrate youth into schools and communities, and omitting mental health supports that could interrupt cycles of violence. When firearm injuries are pathologized as the natural consequence of criminal activity, rather than as preventable pediatric health crises, the medical system abdicates its responsibility to these children, further exacerbating cycles of violence and poor health outcomes.

The health care system’s response to pediatric firearm injuries stands in stark contrast to its approach to other serious pediatric conditions. While conditions such as asthma and cancer benefit from structured, evidence-based care pathways with clearly defined clinical guidelines maintained by national professional organizations, pediatric firearm injuries lack comparable frameworks (Chapman et al., 2024; Flores, 2010). These established pathways integrate interdisciplinary teams, standardized protocols, consistent follow-up schedules, and comprehensive support services. In contrast, up to 69% of firearm injury survivors are lost to follow-up after hospital discharge, despite evidence that follow-up care that addresses physical recovery and behavioral health needs through interdisciplinary services improves outcomes (Aboutanos et al., 2011; Limbos et al., 2007; Prescher et al., 2023).

These disparities reflect multiple intersecting factors. First, firearm violence has historically been framed as a criminal justice issue rather than a public health problem, discouraging medical institutions from developing systematic approaches (Sakran et al., 2020). Second, firearm injuries lack a designated medical specialty or governing body to champion guideline development (Sakran et al., 2022). Pulmonology owns asthma care; oncology owns cancer care, but firearm injuries fall between emergency medicine, trauma surgery, primary care, and behavioral health without clear ownership or accountability. Third, political barriers have constrained the research funding needed to generate an evidence base. The Dickey Amendment, passed in 1996, effectively halted federally funded firearm injury research for over two decades, creating knowledge gaps that persist today (Rostron, 2018). Even as research funding has resumed, it remains inadequate relative to the burden of disease. Pediatric firearm injury research receives only 3.3% of the funding that would be predicted based on mortality burden—requiring a 30-fold increase to be commensurate with other leading causes of pediatric death (Cunningham et al., 2019). This chronic underfunding has left researchers without the data infrastructure, longitudinal cohort studies, and intervention trials needed to develop evidence-based guidelines comparable to those for asthma and cancer. Fourth, reimbursement structures create financial disincentives for hospitals to invest in comprehensive firearm injury care. While acute trauma care is generally reimbursed, the follow-up services most critical for long-term recovery—including behavioral health care, care coordination, rehabilitation, and community-based violence intervention programs—are often inadequately covered by insurance or not billable at all (Simpson et al., 2022). Without sustainable revenue streams to support multidisciplinary teams, longitudinal follow-up clinics, and violence intervention specialists, hospitals struggle to justify the upfront investment required to build comprehensive programs, even when institutional leaders recognize the need. This creates a problematic cycle: lack of reimbursement prevents program development, which perpetuates fragmented care and poor outcomes. Finally, implicit biases within health care systems—manifesting through differential treatment, lower expectations for recovery, and assumptions about patient culpability—undermine institutional motivation to invest in comprehensive care models for populations disproportionately affected by firearm violence (FitzGerald & Hurst, 2017; Hall et al., 2015). Research demonstrates that health care providers hold implicit biases against racial and ethnic minority patients that negatively affect clinical decision-making, treatment recommendations, and patient-provider interactions (Hall et al., 2015). These biases are particularly pronounced in pain management, with studies showing that Black youth receive inadequate pain treatment compared to White youth for identical conditions (Goyal et al., 2015). When these pervasive biases intersect with the stigma attached to firearm injury and assumptions about culpability, the quality gap widens further, contributing to lower institutional investment in care pathways for this patient population.

Addressing these disparities requires structural reforms that dismantle the policies and practices perpetuating inequity, alongside clinical guidelines that explicitly center equity and counteract historical marginalization (Murry et al., 2023). These barriers can be effectively addressed. For example, other pediatric conditions once lacked coordinated care models until professional organizations, parent advocates, and policymakers collaborated to establish them. The development of comprehensive clinical guidelines for pediatric firearm injuries represents a necessary step toward achieving equity in care delivery.

The objectives of this work are threefold: first, to identify specific gaps in pediatric firearm injury care by examining how current fragmented practices compare to established chronic illness management models; second, to highlight the systemic and structural inequities that contribute to disparate treatment, including implicit biases in hospital settings and broader societal perceptions of firearm violence; and third, to propose clear, evidence-informed clinical guidelines that reduce these disparities and ensure firearm-injured youth receive comprehensive, coordinated care comparable to children with other serious health conditions.

## Methods

### Literature Review

To identify current clinical practice guidelines for pediatric chronic illnesses, we conducted a pragmatic scan of the literature and practice landscape. We performed iterative PubMed searches using combinations of terms including: (“firearm injury” OR “gun violence” OR “gunshot wound”) AND (“pediatric” OR “children” OR “adolescent”) AND (“clinical guidelines” OR “clinical pathways” OR “care model” OR “intervention” OR “prevention” OR “follow-up care”). We prioritized publications from the past decade while including foundational studies. We also reviewed published guidelines for pediatric asthma management (National Heart, Lung, and Blood Institute; Global Initiative for Asthma), pediatric cancer care (Children’s Oncology Group; National Cancer Institute), and pediatric trauma care (American College of Surgeons; American Academy of Pediatrics). To ensure comprehensive coverage of both published and grey literature, we supplemented database searches with informal consultations among clinical colleagues and pediatric subspecialty experts, who identified guidelines in active use, emerging recommendations, and resources not readily accessible through traditional search methods.

We considered comparing firearm injuries to other acute injury types such as motor vehicle crashes or falls. However, while these comparisons could illuminate disparities in trauma care delivery, they would not address the fundamental gap we seek to highlight: firearm injuries—despite causing higher mortality and morbidity than other pediatric injury mechanisms and creating substantial long-term physical and psychological sequelae—are not afforded the same systematic, sustained, interdisciplinary care frameworks that exist for other serious pediatric health conditions (Ranney et al., 2019; Wolf et al., 2019). Our intent is not merely to benchmark firearm injury care against other acute injuries, but rather to demonstrate that firearm violence constitutes a preventable, chronic public health crisis deserving of the same institutional commitment, clinical rigor, and comprehensive support systems that health care has successfully developed for conditions like asthma and cancer. We therefore chose these conditions to serve as comparators because they require the following: integration across multiple care settings and coordination among diverse specialists; adherence to evidence-based guidelines maintained by national professional organizations; family engagement as central to treatment outcomes; attention to psychosocial dimensions of illness; and longitudinal frameworks for monitoring recovery and supporting transition to adulthood. Critical differences do exist: firearm injuries can occur abruptly without opportunity for screening; spread through peer and neighborhood networks, creating ongoing safety concerns; and may involve legal system involvement, social stigma, and retaliation risk not experienced with chronic diseases. These differences underscore the need to adapt—not simply replicate—chronic care models to address the unique contextual factors surrounding firearm injuries.

### Conceptual Framework Application

We applied the Injury Equity Framework (Kendi & Macy, 2023) as an organizing structure for guideline development. This framework emphasizes multilevel determinants of injury and recovery, directing attention to structural factors including racism, economic marginalization, and policy decisions shaping both injury risk and care access. The framework guided our approach to ensuring that proposed guidelines explicitly address equity rather than assuming that standardized care automatically produces equitable outcomes.

## Results

Our review of the literature identified well-established clinical pathways for pediatric asthma and cancer care but revealed a stark absence of comparable standardized approaches for pediatric firearm injuries. The following sections, summarized in Table 1, outline core components of care across the prevention continuum, highlighting gaps in firearm injury management and opportunities to adapt care models for youth recovering from firearm trauma. This comparison reveals not clinical differences in treating the physical condition, but rather systemic differences in how health care institutions organize care, allocate resources, ensure accountability, and support families facing serious pediatric health challenges.

**Table 1:** Comparison of pediatric asthma, cancer, and firearm injury care across the spectrum of prevention

Prevention level	Pediatric asthma	Pediatric cancer	Pediatric firearm injuries	Guidelines and standards
Primary	Reduce environmental triggers through home-based interventions (NHLBI Asthma Management Guidelines, GINA Guidelines) Routine well-child visits with risk assessment and patient/family education on asthma management Promotion of inhaler technique education and adherence strategies	Genetic screening and counseling for high-risk populations (American Cancer Society Pediatric Cancer Prevention Guidelines) Public health initiatives to reduce environmental and carcinogenic exposures in children Early detection protocols through routine pediatric assessments	Limited firearm safety counseling in pediatric settings due to lack of universal guidelines American Academy of Pediatrics recommends secure firearm storage discussions with parents, though not standardized across health care settings Few systemic policy-driven initiatives for firearm injury prevention in clinical practice	**Asthma:** NHLBI Asthma Management Guidelines, GINA Guidelines **Cancer:** ACS Pediatric Cancer Prevention Guidelines **Firearm injuries:** No universally accepted guidelines
Secondary	Early identification of exacerbations and use of stepwise medication adjustments (NIH Asthma Care Quick Reference) Personalized asthma action plans, including symptom recognition and emergency response steps Coordination with school nurses and community-based programs to reinforce management strategies	Rapid diagnostic pathways and standardized chemotherapy, radiation, and surgical intervention protocols (National Cancer Institute Pediatric Treatment Guidelines) Interdisciplinary team-based treatment plans with psychosocial support integration Emphasis on early supportive care, including nutrition and pain management to improve treatment adherence	Trauma care guided by ACS Trauma Care Standards, though inconsistent for pediatric firearm injuries Variable hospital-based screening for psychosocial risk factors and mental health needs post-injury No established national protocols for firearm injury rehabilitation or outpatient transition plans	**Asthma:** NIH Asthma Care Quick Reference **Cancer:** National Cancer Institute Pediatric Treatment Guidelines **Firearm injuries:** ACS Trauma Care Standards (inconsistent for pediatrics)
Tertiary	Long-term controller therapy with inhaled corticosteroids, biologics, or leukotriene modifiers for severe cases Regular follow-ups with pulmonary specialists and integration of school-based asthma programs Home visits for high-risk patients to address environmental contributors and reinforce adherence (School-Based Asthma Management Programs	Long-term survivorship monitoring for late effects of treatment, including secondary malignancies and organ dysfunction Psychosocial rehabilitation programs and structured transition-to-adulthood care (Children’s Oncology Group Survivorship Guidelines) Coordination of care across primary and specialty providers to ensure ongoing cancer surveillance	No standardized follow-up care pathways for pediatric firearm injury survivors Limited integration of long-term mental health support or rehabilitation services Lack of structured reintegration programs for education, employment, and social support post-injury	**Asthma:** School-Based Asthma Management Programs **Cancer:** Children’s Oncology Group Survivorship Guidelines **Firearm injuries:** No standardized follow-up guidelines

*NHLBI* National Heart, Lung, and Blood Institute; *GINA *Global Initiative for Asthma, *NIH* National Institute of Health, *ACS* American Cancer Society

Asthma and cancer are managed through structured, interdisciplinary approaches that emphasize long-term care coordination. Asthma care typically involves pediatricians, pulmonologists, and allergists collaborating with school nurses and community health workers to provide routine follow-ups, medication adjustments, and environmental assessments (NHLBI,  2020; GINA, 2023). Similarly, pediatric cancer treatment includes a team of oncologists, surgeons, radiologists, psychologists, and social workers, with survivorship clinics playing a crucial role in ongoing surveillance and monitoring (Nass & Patlak, 2015; Children’s Oncology Group Survivorship Guidelines, 2025). For both conditions, social work and case management consultations have been shown to improve care coordination, reduce emergency department visits and hospitalizations, and address psychosocial and financial needs (Gill et al., 2022; Verulava et al., 2019). In contrast, firearm injuries are often treated as acute events by emergency physicians and trauma surgeons, with limited focus on long-term rehabilitation or psychosocial recovery (Lee et al., 2022). Although some patients are referred to mental health or rehabilitation services, follow-up care is inconsistent and often dependent on individual hospitals’ resources rather than standardized protocols (Prescher et al., 2023). It remains unclear whether youth with firearm injuries consistently receive social work or case management consultations, as these pathways have not been systematically studied or linked to outcomes in this population. Given the complex interplay of trauma, socioeconomic stressors, and community violence surrounding firearm injuries, introducing supportive services must be approached with sensitivity to avoid perceptions of blame or failure among patients and families (Timmer‑Murillo et al., 2023).

The frequency and structure of follow-up care also vary significantly among these conditions. Asthma patients usually have scheduled check-ups every 3 to 6 months, with additional visits for exacerbations (Avery et al., 2021). Cancer patients adhere to structured treatment schedules involving chemotherapy, radiation, and long-term surveillance, often supported by financial and psychological services (Deegan et al., 2023; Nass & Patlak, 2015; Children’s Oncology Group Survivorship Guidelines, 2025; ACS, 2023). Conversely, firearm injury survivors may receive an initial trauma follow-up, but subsequent care is often reactive, with re-engagement occurring primarily in response to complications (Degli Esposti et al., 2022; Sakran et al., 2022). Innovative models like the Trauma Quality of Life (TQoL) Clinic have emerged to address these gaps, providing coordinated care through interdisciplinary teams that include trauma providers, psychologists, physical therapists, social workers, and violence interrupters to support comprehensive recovery (Brandolino et al., 2024).

Parental involvement is another key factor in the management of these conditions. Asthma and cancer care rely heavily on parents as primary caregivers, ensuring medication adherence, attending medical appointments, and advocating for their children’s needs (Nass & Patlak, 2015). These roles are reinforced by caregiver education programs and structured provider engagement (Chang, 2012; Lin et al., 2024; Nass & Patlak, 2015). However, parents of firearm-injured youth often lack clear guidance on how to support their child’s long-term recovery, as there are no formalized pathways for their involvement (Sakran et al., 2020). Without standardized counseling or follow-up care, families may struggle to navigate rehabilitation, mental health support, and school reintegration, contributing to poorer outcomes.

Beyond direct medical care, external systems play a crucial role in shaping patient outcomes (Perkins & Graham-Bermann, 2012). Asthma and cancer patients benefit from strong integration with schools, housing programs, and social services. Schools actively support asthma management through medication administration and emergency planning, while cancer survivors receive assistance from advocacy networks that provide funding, counseling, and community reintegration support (Deegan et al., 2023; Scheckner et al., 2015). Firearm-injured youth, by contrast, may face systemic barriers such as legal scrutiny, social stigma, and inconsistent access to recovery resources (Sakran et al., 2020). The absence of structured reintegration programs can complicate school reentry and social adjustment, further exacerbating disparities.

Insurance coverage further compounds these disparities. Asthma and cancer treatments are generally covered, though some families encounter barriers to accessing specialty care or long-term rehabilitation. Cancer care—including chemotherapy and hospital stays—is typically reimbursed, though out-of-pocket costs may be high. In contrast, while emergency care for firearm injuries is usually covered, follow-up services like rehabilitation and behavioral health care are often inadequately reimbursed, limiting access to essential recovery supports (Simpson et al., 2022). This results in many patients forgoing essential post-injury care, widening the gap between firearm injury recovery and chronic disease management.

Together, these differences illustrate systemic inequities in care delivery. Asthma and cancer have well-established, long-term care pathways that emphasize ongoing management, structured follow-up, and community integration. Firearm injuries, however, are often managed as isolated incidents, with little emphasis on long-term recovery or reintegration. Addressing these gaps requires reimagining how firearm injuries are conceptualized within health care, ensuring that survivors receive the same level of coordinated care, psychosocial support, and systemic integration as children with chronic illnesses.

## Recommendations for a Standardized Pediatric Firearm Injury Care Guideline

**Fig. 1:**
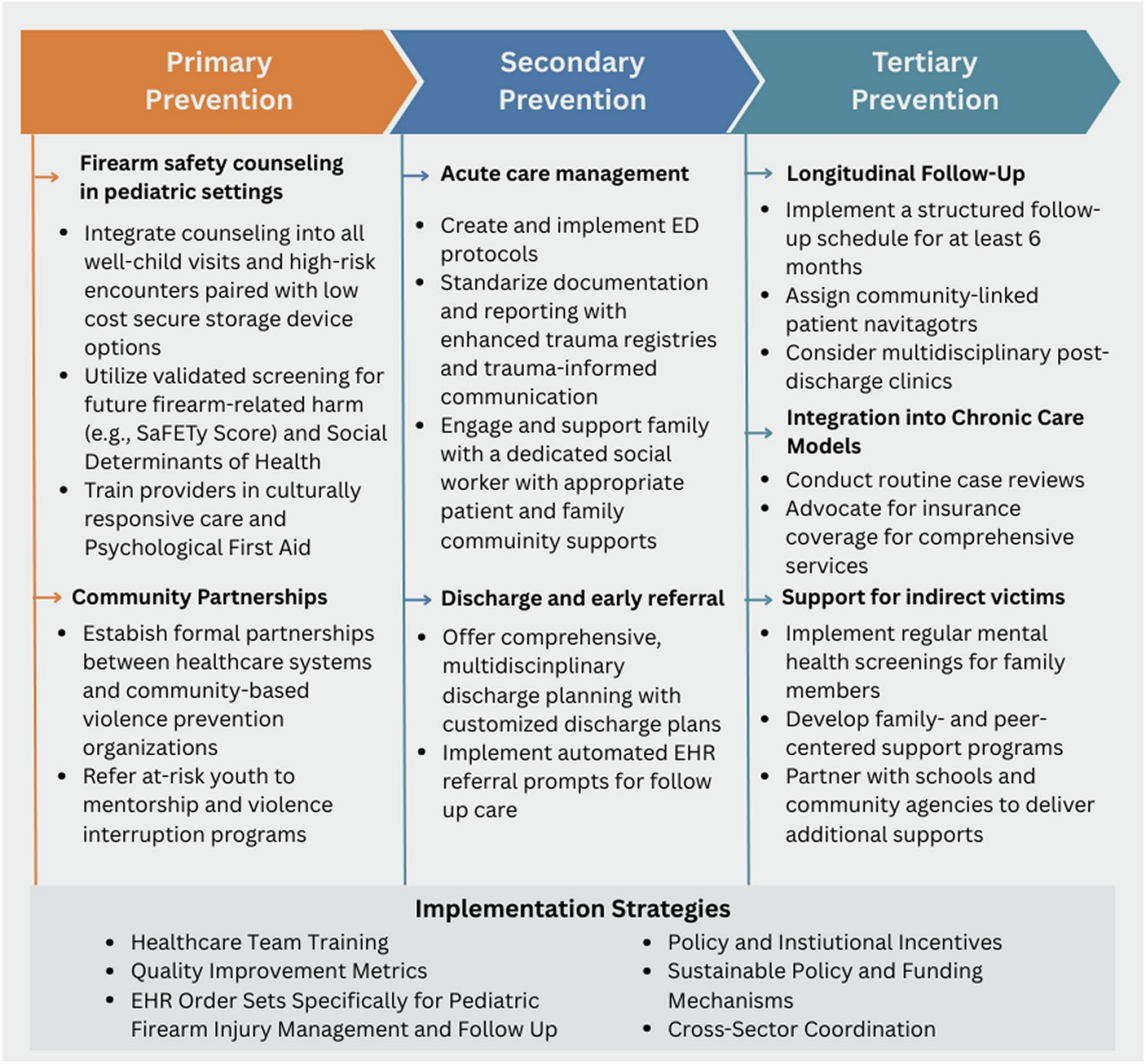
Proposed clinical guidelines for pediatric firearm injury prevention across the prevention spectrum. Note. SaFETy, serious fighting, peer weapon carrying, community involvement, firearm threats; ED, emergency department; EHR, electronic health record

Despite the urgent need for standardized pediatric firearm injury guidelines, care remains fragmented and inconsistent. Unlike chronic conditions such as asthma and cancer, which benefit from coordinated, interdisciplinary care pathways, firearm injuries lack a designated specialty or governing body to oversee comprehensive clinical management—contributing to inconsistent treatment and disparities in long-term outcomes (Sakran et al., 2022). To address this gap, we propose evidence-informed guidelines grounded in the Injury Equity Framework, aiming to establish a cohesive, interdisciplinary approach to pediatric firearm injury care (Fig. 1). The following section proposes clinical guidelines designed to bring the same rigor, comprehensiveness, and family-centeredness to firearm injury care that currently exists for other conditions.

### The Injury Equity Framework: A Model for Pediatric Firearm Injury Care

The Injury Equity Framework provides a conceptual foundation for understanding how intersecting forms of oppression—such as racism, classism, and ableism—shape injury risk, severity, and recovery across structural and individual levels (Kendi & Macy, 2023). Grounded in the Haddon Matrix, it incorporates multilevel influences and timelines of injury to guide the development of equity-driven interventions. The following recommendations draw on this framework and align with guidance from professional societies including the American Academy of Pediatrics (AAP), the American Pediatric Surgical Association (APSA), and the American College of Surgeons (ACS) (Lee et al., 2022).

### Primary Prevention and Early Intervention: Upstream Interventions to Prevent Injury

Primary prevention is a cornerstone of reducing pediatric firearm injuries, focusing on early identification of risk and addressing upstream factors before harm occurs. Firearm safety counseling is an evidence-based strategy to improve household secure storage and is recommended to be integrated into all well-child and injury or mental health-related visits (Lee et al., 2022; Rowhani-Rahbar et al., 2016). This includes the use of validated screening tools such as the SaFETy Score—which assesses fighting, peer weapon access, environmental risk, and firearm threats—as well as screening for social drivers of health (SDoH) with embedded community referrals (Bonne & Dicker, 2020; Goldstick et al., 2017). Providers should be trained in culturally responsive firearm safety counseling, particularly for families affected by community violence or for whom firearm ownership is a cultural norm (Betz & Wintemute, 2015). Hospitals should offer or partner with organizations that provide free or subsidized firearm locking devices and refer families to secure firearm storage programs (Freire et al., 2023; Lee et al., 2022). To work towards comprehensive preventative care, hospitals should also partner with community-based violence prevention organizations, schools, and local agencies, and create internal programs addressing key SDoH such as housing instability and food insecurity (Strong et al., 2025). Youth identified as being particularly vulnerable should be referred to evidence-based community programs including mentorship, family-based therapy, and violence interruption services (Cheng et al., 2008; Limbos et al., 2007).

### Secondary Prevention: Standardized Holistic Trauma Care

Secondary prevention begins with standardized, trauma-informed care in the emergency department. Trauma team activation should be based on clinical acuity, with automatic consultations with behavioral health, social work, and case management. Initial stabilization should follow Advanced Trauma Life Support (ATLS) protocols while integrating trauma-informed communication. Within 24 h of admission, all firearm-injured patients should undergo psychosocial screening using validated tools assessing acute stress, adjustment symptoms, and risk factors such as intimate violence, housing insecurity, and firearm access (Lee et al., 2022). Hospitals should incorporate standardized electronic health record (EHR) templates and validated SDoH screeners to support consistent documentation (Smith et al., 2024). These assessments should be integrated with hospital-based violence intervention programs (HVIPs), which connect patients to trauma-informed interdisciplinary services and improve outcomes (Bonne & Dicker, 2020). Each family should be assigned a trauma-informed social worker within 24 h to initiate mental health support and referrals to grief counseling (Dishion et al., 2003). In cases related to community violence, community partners should be introduced early to provide culturally responsive care (Aboutanos et al., 2011).

Following stabilization, interdisciplinary discharge planning must include the patient’s family and key care team members. Plans should include follow-up appointments, referrals to rehabilitation and mental health services, and school reentry support. Extended case management should help coordinate care and link families to economic, legal, and housing services. Automated EHR referral prompts should direct patients to trauma-focused behavioral health services, peer support, and school-based or culturally relevant counseling. This proactive approach to coordinated care ensures that youth receive comprehensive support throughout early recovery, mitigating the risk of long-term psychological and functional impairments.

### Tertiary Prevention: Long-Term Recovery and Reintegration

Recognizing firearm injury as a chronic condition is essential for sustainable recovery. Survivors often experience prolonged physical limitations, psychological sequelae, and social disruption, necessitating an ongoing, interdisciplinary model of care (Ranney et al., 2019; Vella et al., 2020). A standardized longitudinal follow-up schedule should be implemented, with clinical visits at approximately 1, 3, and 6 months post-discharge, and flexibility for extended care as clinically indicated (Jacobson et al., 2025). Return-to-activity plans should include physical, psychological, and legal considerations. Where possible, interdisciplinary post-discharge clinics—such as the Trauma Quality of Life (TQoL) Clinic—should coordinate medical, behavioral health, rehabilitation, and social services (Brandolino et al., 2024).

Recovery models must also address indirect victims. The intergenerational and interpersonal transmission of trauma following firearm violence extends far beyond the injured individual, creating cascading risks throughout family and peer networks. Vicarious exposure to firearm violence—including witnessing shootings or having close friends or family members injured or killed—significantly increases risk for both future victimization and perpetration (Bingenheimer et al., 2005; Fowler et al., 2009). Every additional exposure to someone who has sustained a gunshot wound predicts increased likelihood of subsequent firearm involvement (Papachristos et al., 2015; Song et al., 2023). Post-traumatic stress symptoms—including hypervigilance, distrust, anger, and heightened threat perception—can motivate aggressive or provocative behaviors that increase risk of violent retaliation or re-injury (Mehari et al., 2025). Youth experiencing untreated PTSD may respond disproportionately to minor provocations or engage in preemptive aggression driven by fear. Moreover, firearm violence exhibits contagion effects within social networks. Peer firearm carrying—often a response to fear following violence exposure—increases individual risk for both victimization and perpetration, creating self-reinforcing cycles where protective behaviors paradoxically elevate danger (Dijkstra et al., 2012; Mehari et al., 2025). Current hospital-based victim services typically focus exclusively on the injured individual, missing critical opportunities to interrupt violence transmission. Trauma services should extend to siblings and peers at risk of secondary trauma. Family members and high-risk youth should be offered regular mental health screening and referrals. Siblings should receive age-appropriate trauma screening and access to evidence-based treatments. Peers identified as witnesses or close contacts should be connected to school-based counseling and community violence prevention programs. Addressing the full spectrum of trauma across family and peer networks can help interrupt cycles of retaliation and contagion, promoting resilience and long-term recovery for firearm-injured youth, their families, and their communities.

## Discussion

Disparities in pediatric health care are well-documented, particularly among racially and socioeconomically marginalized children, and are especially pronounced in the context of firearm injuries. Black and Latino youth experience disproportionately high injury rates, reduced access to follow-up care, and limited engagement with psychosocial services (Sakran et al., 2020). These disparities are compounded by implicit bias within health care systems and a pervasive tendency to frame firearm injuries as legal or disciplinary issues rather than public health concerns—both of which undermine provider empathy and investment in post-injury care. Unlike chronic pediatric conditions such as asthma or cancer, which benefit from structured, interdisciplinary care models, firearm injuries are often treated as isolated events without standardized protocols for long-term recovery. This pattern reflects a systemic failure to provide comprehensive care for a population facing substantial physical, psychological, and economic burdens—and underscores the urgent need for standardized, trauma-informed pathways to support equitable recovery.

Established care models for chronic pediatric conditions such as asthma and cancer provide critical insight into how interdisciplinary, family-centered approaches can improve long-term outcomes. Like firearm injury, these conditions require integration of behavioral health services, care coordination, and sustained family engagement. However, firearm injuries differ in important ways: they occur abruptly, are often tied to systemic violence, and can destabilize households and communities. Unlike chronic conditions that allow for anticipatory guidance and structured adaptation, firearm injuries demand immediate crisis response and long-term support without warning. Additionally, firearm violence may propagate through peer and neighborhood networks, mirroring infectious disease transmission more than isolated clinical episodes. As such, while chronic care models offer a foundation, firearm injury interventions must be tailored to address the acute, destabilizing, and socially embedded nature of this trauma.

Persistent disparities in pediatric firearm injury care demand structural solutions. Using the Injury Equity Framework, a model that emphasizes systemic drivers of injury and recovery and shifts focus from individual behavior to the broader sociopolitical and economic contexts in which firearm violence occurs, we developed a set of clinical guidelines for pediatric firearm injury care that span primary, secondary, and tertiary prevention (Kendi & Macy, 2023). At the primary prevention level, guidelines call for universal firearm safety screening during pediatric visits, integrated with anticipatory guidance and referrals to local violence prevention and support services. Secondary prevention efforts emphasize standardized protocols for acute trauma care, including timely psychosocial assessments and automatic referrals to interdisciplinary support teams. Finally, tertiary prevention addresses the long-term needs of firearm-injured youth and their families through structured follow-up care, individualized recovery plans, and sustained behavioral health and case management support. By embedding these practices within hospital systems, pediatric care can evolve to more effectively meet the enduring medical and social needs of children impacted by firearm violence.

### Implementing and Sustaining the Guidelines

Translating these guidelines into lasting change requires coordinated action across multiple levels. Workforce development must include mandatory trauma-informed care training for all clinicians and staff, incorporating modules on implicit bias for continuing medical education credit, firearm safety counseling, and equity frameworks. Clinical workflows should integrate standardized order sets that automate trauma team activation, initiate validated psychosocial screening, prompt referrals to social work and community services, and schedule follow-up at 1, 3, and 6 months post-discharge. Hospitals must establish EHR-based tracking systems and firearm injury dashboards to monitor key process measures—including referral completion, psychosocial service engagement, readmissions, reinjury rates, and follow-up adherence—while enhancing trauma registry reporting to capture prior violence exposure and social determinants of health. Routine case review conferences modeled after oncology care should assess progress and inform care modifications. Institutional accountability mechanisms should include hospital accreditation standards requiring firearm injury prevention protocols and post-discharge pathways, with hospital-based violence intervention programs (HVIPs) mandated at all Level 1 pediatric trauma centers.

Sustainability depends on policy reforms that expand Medicaid and commercial insurance reimbursement for mental health care, rehabilitation, and community-based services, ensuring durable funding for prevention and longitudinal care teams. A national pediatric organization (e.g., AAP or APSA) should lead guideline development and maintenance, while federal and state funding supports hospital-community partnerships and rigorous evaluation of equity-focused interventions.

Violence prevention requires coordination across healthcare, education, criminal justice, social services, and community organizations, with hospitals serving as one component of a comprehensive ecosystem rather than sole solution providers. Hospitals should establish formal partnerships with schools, housing authorities, workforce development programs, and legal aid organizations to address social determinants of health affecting recovery. These strategies create the infrastructure needed to move beyond episodic, crisis-driven responses toward a sustainable, equity-centered model delivering the interdisciplinary, long-term support that children and families affected by firearm violence deserve.

Firearm injury is not an outlier in pediatric care—it is a prevalent, preventable, and structurally patterned public health crisis. It must be addressed with the same strategic investment, clinical rigor, and institutional commitment afforded to other serious pediatric conditions. With these systems in place, pediatric trauma care can evolve from fragmented, crisis-based treatment to a comprehensive, sustainable, and equity-centered model.

## Limitations and Future Directions

These proposed guidelines, while grounded in existing literature and informed by successful models in other conditions, have not been formally validated through multi-site implementation studies. Prospective research is essential to determine which guideline components most effectively improve outcomes, which implementation strategies achieve highest adoption rates, and how guidelines should be adapted for diverse contexts including rural settings, community hospitals with limited subspecialty resources, and regions with different patterns of firearm violence (e.g., predominantly unintentional injuries in younger children vs. intentional interpersonal violence in adolescents). Additionally, guidelines require ongoing updating as new research emerges. Professional organizations assuming ownership of guideline maintenance must establish processes for regular literature review, stakeholder input including community voices, and evidence-based revisions. This iterative process, standard for asthma and cancer guidelines, ensures recommendations remain current and responsive to emerging challenges. Finally, guidelines alone are insufficient. They must be accompanied by structural reforms addressing root causes of violence including poverty, educational inequity, housing instability, and discriminatory policing practices. Health care institutions cannot solve community violence independently but must partner with schools, housing authorities, criminal justice reform advocates, and community-based organizations working toward systemic change. This requires humility about medicine’s limitations and commitment to long-term collaborative efforts extending beyond clinical walls.

## Conclusions

Pediatric firearm injuries are not isolated incidents—they are sentinel events that reflect deep systemic failures and demand a coordinated clinical and public health response. Firearm-injured youth, who are disproportionately Black and socioeconomically marginalized, receive fragmented care that pales in comparison to the structured, interdisciplinary approaches afforded to children with asthma or cancer. These disparities are not coincidental; they stem from long-standing disinvestment and a lack of standardized clinical guidance. By adopting trauma-informed, equity-centered guidelines that span the continuum of care and aligning implementation strategies with proven systems-change levers, hospitals can move from reactive treatment to proactive, accountable care. Ensuring that every child who survives a firearm injury has access to comprehensive medical, psychosocial, and community-based support is not only feasible—it is a moral and medical imperative.

## Supplementary Information

Below is the link to the electronic supplementary material.

ESM 1 (20.6 KB DOCX)

## Data Availability

All data and information used in this manuscript are publicly available. No primary data were colleted for this study.
