# Supplementary material for: Disparities in Pediatric Firearm Injury Care: A Comparison of Chronic Illness Pathways
Source: Prev Sci. 2026 Jan 2;27(5):737–49. doi: 10.1007/s11121-025-01865-0 (PMC13421351; doi:10.1007/s11121-025-01865-0)
Supplement: Supplementary file 1 — (20.6 KB DOCX) [file 11121_2025_1865_MOESM1_ESM.docx]

**Expanded Supplement: Search Strategy and Guideline Sources**

**Search Details**

| Element | Description |
| --- | --- |
| Database searched | PubMed |
| Search terms | ("firearm injury" OR "gun violence") AND ("pediatric" OR "children" OR "adolescent") AND ("guidelines" OR "policy statement" OR "clinical recommendations" OR "prevention" OR "care model") |
| Filters applied | Publication date: last 10 years; Population: children (0–19 years) |
| Date range | July 2015 – August 2025 |
| Date of last search | October 15, 2025 |
| Additional sources reviewed | - **Firearm Injury Prevention**:   - AAP Policy Statement: *Firearm-Related Injuries and Deaths in Children and Youth: Injury Prevention and Harm Reduction* (2022)   - APSA Position Statement: *Firearm Injuries and Children* (2019)   - AAP Technical Report on Firearm Injuries - **Pediatric Surgery / Trauma**:   - ACS Trauma Quality Program Best Practices   - AAP Policy: *Management of Pediatric Trauma*   - Pediatric Trauma Society Guidelines - **Asthma Guidelines**:   - NHLBI Asthma Management Guidelines (2020 Focused Updates)   - Global Initiative for Asthma (GINA) 2024/2025 Reports - **Oncology Guidelines**:   - Children’s Oncology Group Supportive Care Guidelines   - COG Long-Term Follow-Up Guidelines (Version 6.0, 2023)   - NCI PDQ Pediatric Cancer Treatment Summaries |

**Results of search**

- 475 PubMed articles screened
- 28 relevant articles included
- Plus 10 major guidelines/policy documents from AAP, APSA, ACS, NHLBI, GINA, COG, and NCI

We conducted a PubMed search using the terms (“firearm injury” OR “gun violence”) AND (“pediatric” OR “children” OR “adolescent”) AND (“guidelines” OR “policy statement” OR “clinical recommendations” OR “prevention” OR “care model”) Filters were applied to restrict results to publications within the last 10 years and populations aged 0–19 years. This **search yielded 475 relevant articles**, which we supplemented by reviewing authoritative policy statements and technical reports from professional organizations, including the American Academy of Pediatrics (AAP), American College of Surgeons (ACS), and American Pediatric Surgical Association (APSA). We also incorporated evidence-based reviews and frameworks addressing firearm injury prevention, hospital-based violence intervention programs, and post-discharge care for firearm injury survivors. Ultimately, **28 references were identified** as directly relevant to pediatric firearm injury care delivery and follow-up management.

Guidelines for pediatric asthma and cancer care are published and maintained by leading specialty organizations and are widely recognized as the standard of care. For asthma, we relied on the National Heart, Lung, and Blood Institute (NHLBI) 2020 Focused Updates and the Global Initiative for Asthma (GINA) strategy reports. For pediatric oncology, we reviewed the Children’s Oncology Group (COG) Long-Term Follow-Up Guidelines and National Cancer Institute (NCI) PDQ summaries. These resources are centralized, regularly updated, and explicitly designed to guide clinical practice, making the process of locating and synthesizing asthma and cancer guidelines significantly simpler compared to the fragmented and less standardized landscape of pediatric firearm injury care.

*Firearm Injury Care Articles*

1. Aboutanos et al. (2011) – Brief violence interventions with community case management services are effective for high-risk trauma patients.
2. Betz & Wintemute (2015) – Physician counseling on firearm safety: A new kind of cultural competence.
3. Bingenheimer et al. (2005) – Firearm violence exposure and serious violent behavior.
4. Bonne & Dicker (2020) – Hospital-based violence intervention programs to address social determinants of health and violence.
5. Brandolino et al. (2024) – Improved follow-up care for gun violence survivors in the Trauma Quality of Life Clinic.
6. Cheng et al. (2008) – Effectiveness of a mentor-implemented violence prevention intervention for assault-injured youth presenting to the emergency department.
7. Degli Esposti et al. (2022) – Firearm injuries are a critical driver of health disparities in the United States.
8. Formica (2021) – An eye on disparities, health equity, and racism—The case of firearm injuries in urban youth.
9. Fowler et al. (2017) – Childhood firearm injuries in the United States.
10. Gastineau et al. (2024) – Health care utilization after nonfatal firearm injuries.
11. Goldstick et al. (2017) – Development of the SaFETy Score: A clinical screening tool for predicting future firearm violence risk.
12. Jacobson et al. (2025) – Understanding and promoting equitable post-discharge care in firearm injury survivors.
13. Lee et al. (2022) – Firearm-related injuries and deaths in children and youth: Injury prevention and harm reduction.
14. Limbos et al. (2007) – Effectiveness of interventions to prevent youth violence: A systematic review.
15. Mehari et al. (2025) – Social-ecological correlates of involvement in firearm-related violence.
16. Mehranbod et al. (2022) – Historical redlining and the epidemiology of present-day firearm violence.
17. Papachristos et al. (2015) – The social contagion of nonfatal gunshot injuries.
18. Prescher et al. (2023) – Trauma clinic follow-up: Predictors of nonattendance and patient-reported reasons for no show.
19. Ranney et al. (2019) – Long-term consequences of youth exposure to firearm injury, and how do we prevent them?
20. Rowhani-Rahbar et al. (2016) – Effectiveness of interventions to promote safe firearm storage.
21. Sakran et al. (2020) – Pediatric firearm injuries and fatalities: Do racial disparities exist?
22. Sakran et al. (2022) – Coordinating a national approach to violence prevention.
23. Smith et al. (2024) – Addressing social determinants of health may improve emergency department utilization after firearm violence.
24. Song et al. (2023) – Firearm injuries in children and adolescents: Health and economic consequences among survivors and family members.
25. Strong et al. (2025) – Community-based hospital violence intervention programs: Current best practices and future directions.
26. Timmer Murillo et al. (2023) – Comprehensive framework of firearm violence survivor care: A review.
27. Vella et al. (2020) – Long-term functional, psychological, emotional, and social outcomes in survivors of firearm injuries.
28. Wolf et al. (2019) – Evaluation of injury severity and resource utilization in pediatric firearm and sharp force injuries.
